# Use of gene therapy for optic nerve protection: Current concepts

**DOI:** 10.3389/fnins.2023.1158030

**Published:** 2023-04-06

**Authors:** Kexin Xu, Lu Yu, Zhiyi Wang, Pei Lin, Ningzhi Zhang, Yiqiao Xing, Ning Yang

**Affiliations:** ^1^Department of Ophthalmology, Renmin Hospital of Wuhan University, Wuhan, Hubei, China; ^2^Department of Ophthalmology, Aier Eye Hospital of Wuhan University, Wuhan, Hubei, China

**Keywords:** optic nerve injury, gene therapy, CRISPR, neuroprotection, retinal ganglion cell

## Abstract

Gene therapy has become an essential treatment for optic nerve injury (ONI) in recent years, and great strides have been made using animal models. ONI, which is characterized by the loss of retinal ganglion cells (RGCs) and axons, can induce abnormalities in the pupil light reflex, visual field defects, and even vision loss. The eye is a natural organ to target with gene therapy because of its high accessibility and certain immune privilege. As such, numerous gene therapy trials are underway for treating eye diseases such as glaucoma. The aim of this review was to cover research progress made in gene therapy for ONI. Specifically, we focus on the potential of gene therapy to prevent the progression of neurodegenerative diseases and protect both RGCs and axons. We cover the basic information of gene therapy, including the classification of gene therapy, especially focusing on genome editing therapy, and then we introduce common editing tools and vector tools such as Clustered Regularly Interspaced Short Palindromic Repeats (CRISPR) -Cas9 and adeno-associated virus (AAV). We also summarize the progress made on understanding the roles of brain derived neurotrophic factor (BDNF), ciliary neurotrophic factor (CNTF), phosphatase-tensin homolog (PTEN), suppressor of cytokine signal transduction 3 (SOCS3), histone acetyltransferases (HATs), and other important molecules in optic nerve protection. However, gene therapy still has many challenges, such as misalignment and mutations, immunogenicity of AAV, time it takes and economic cost involved, which means that these issues need to be addressed before clinical trials can be considered.

## 1. Introduction

### 1.1. Overview of optic nerve injury

Optic nerve injury (ONI), also known as traumatic optic neuritis, is generally an indirect result of injury to the brain or maxillofacial area, although it is also closely related to glaucoma and certain metabolic diseases. ONI is characterized by the loss of retinal ganglion cells (RGCs) and axons, which can lead to an abnormal pupillary light reflex, visual field defects, and even vision loss (Leske, 1983; Tsai, 2013; Zhong, 2016; Kimura et al., 2017). In some cold-blooded vertebrates, ONI leads only to modest RGC death. However, mammals always exhibit near-complete loss of RGC (Liu et al., 2012; Zou et al., 2013).

Given its location in the central nervous system (CNS), damage to the optic nerve is considered irreversible. However, several studies have reported varying degrees of visual recovery in select patients with optic nerve damage (Sosin et al., 2016; Wladis et al., 2021). Thus, there is a possibility that the optic nerve can be regenerated under certain conditions. The aim of this review was to discuss the research progress made on gene therapy for ONI. Gene therapy offers the possibility to rescue neurodegeneration during ONI at a finer level, contributing to the survival and regeneration of RGCs and axons. At the same time, this review also explores in depth the advantages and disadvantages of gene therapy implementation and strives to promote timely delivery of gene therapy to patients.

### 1.2. Inflammation in ONI

Following CNS injury, inflammation occurs as an inevitable step and is usually considered as a negative process because it aggravates nerve damage. However, one lab reported extensive axon regeneration after unintentional injury to the lens, the effects of which could be mimicked by treating intraocular inflammation with a regenerative substance, such as zymosan (Yin et al., 2019). Zymosan can induce the production of a sterile inflammatory response, leading to the aggregation of inflammatory cells such as macrophages and the secretion of nutritional factors, effectively improving the regeneration of RGCs. Another study reported that the pro-regeneration effect of zymosan was attenuated by the knock-out of toll-like receptor 2 (TLR2) and dectin-1, which are expressed on inflammatory cells (Baldwin et al., 2015). These studies indicate that intraocular inflammation may be the key element for axon regeneration.

Despite its negative contributions, inflammation involving macrophages may play a role in promoting optic nerve regeneration. Mannose has been shown to stimulate a moderate amount of axon growth from mature rat RGCs in a cAMP-dependent manner, which can be strongly augmented oncomodulin (Ocm), a protein secreted by activated macrophages (Li et al., 2003). Ocm is secreted by infiltrative neutrophils and macrophages and binds to a high-affinity receptor on RGCs. The small chemokine known as stromal cell-derived factor 1 (SDF1) is another inflammatory factor reported to promote optic nerve regeneration and is also highly expressed in macrophages (Belmadani et al., 2005; Chalasani et al., 2007). Deletion of SDF1 in myeloid cells or its receptor CXCR4 in RGCs reduces optic nerve regeneration that depends on inflammation, while deletion of both Ocm and SDF1 reduces inflammation-induced regeneration by 70–80 (Chalasani et al., 2007).

In addition to the important contribution of macrophages, microglia and astrocytes also play a role in optic nerve regeneration promoted by inflammation. During the regeneration of the optic nerve and in many neurodegenerative diseases such as glaucoma, microglia and astrocytes often exert competing pro-inflammatory and anti-inflammatory effects. Reactive astrocytes can clear cell debris and provide neurotrophic support to neurons; however, A1 astrocytes also exert highly neurotoxic effects (Zamanian et al., 2012; Liddelow et al., 2017). Inhibition of microglial activation results in a significant reduction in neuronal cell death and has been shown to protect RGCs in a rat model of ocular hypertension (Roh et al., 2012; Williams et al., 2016; Salter and Stevens, 2017; Williams et al., 2019). However, ablation of microglia does not appear to greatly improve the survival rate of RGCs, emphasizing that microglia and astrocytes exert both positive and negative effects on optic nerve regeneration (Hilla et al., 2017). As such, further research should focus on how to induce microglia and astrocytes to develop in a direction conducive to the regeneration of RGCs. Different immune cells express different genes depending on the environment and thus play different roles. Scholars have begun to study the transduction of genetic material in immune cells mediated by adeno-associated virus (AAV) vectors, thereby changing the phenotype of immune cells and promoting nerve survival and regeneration.

In addition to the above-mentioned cells, the complement system may also play a role in protecting the optic nerve. The complement cascade responds to pathogens, and complement proteins are also involved in CNS development, neuroplasticity, and other key events. Although many studies have reported detrimental roles of microglia/monocytes (myeloid cells) and complement proteins in the CNS, Gassel et al. (2020) found that complement proteins may be involved in optic nerve repair (Liddelow et al., 2017; Aranda et al., 2019). Using the ONI model in which axons arising from RGCs are disrupted, the authors found that complement proteins C1q and C3, along with microglia expressing the phagocytic complement C3b receptor CR3, were markedly increased, suggesting that all these are necessary for the optic nerve regeneration (Gassel et al., 2020). However, the knockout of Clq, C3, and CR3 attenuates RGC axon regeneration. The classical complement cascade and phagocytic cells may promote axon regeneration by removing myelin debris (Peterson et al., 2021). However, further studies are required to identify the mechanisms by which the complement system can aggravate or attenuate nerve injury.

### 1.3. Glaucoma and ONI

Glaucoma is a common cause of ONI. The loss of RGCs in the early phase of glaucoma is difficult to observe, and only when the thickness of the retinal nerve fiber layer (RNFL) has decreased substantially, leading to optic neuropathy, such loss can be detected by current instruments. Since the apoptosis of RGCs and axons due to ONI is irreversible, identifying the pathogenic mechanisms of ONI and developing strategies for attenuating further injury remain imperative.

In glaucoma, pathologic elevation of intraocular pressure (IOP) is the primary cause of ONI and RGCs death (Weinreb and Khaw, 2004). Elevated IOP directly compresses the lamina cribrosa, blocks axoplasmic transport, reduces neurotrophin intake, and interrupts papillary blood perfusion. Numerous studies have reported associations between genetic mutations and genetic factors with glaucoma (Wiggs and Pasquale, 2017). The *Pro370Leo* mutation of *MYOC* leads to misfolding of the encoded protein, which cannot exit the cell, in turn leading to excessive intracellular accumulation (Wang et al., 2007). Such accumulation triggers endoplasmic reticulum stress and a decrease in the mitochondrial membrane potential in trabecular meshwork cells, thus initiating cell apoptosis. Mutations in *E50K* of *OPTN* can lead to oxidative stress-mediated apoptosis in RGCs and affect aqueous humor production, composition, and effusion by interfering with vesicle-mediated transport, as well as autophagy (Sirohi and Swarup, 2016).

### 1.4. Traumatic optic neuropathy (TON)

Traumatic brain injury (TBI) is the leading cause of ONI. ONI due to TBI has been associated with increased levels of glial fibrillary acidic protein (GFAP), ubiquitin carboxy-terminal hydrolase L1 (UCH-L1), and neurofilament light chain protein (NFL). Given the characterization of the optic nerve as a component of the central nervous system and its proximity to the brain itself, TON can be viewed as a focal form of TBI.

In one previous study, the authors measured levels of GFAP, UCH-L1, and NFL immediately before the optic nerve crush and 1 h post-injury in 10 Yucatan minipigs (Bramblett et al., 2021). Increases in levels of all three proteins were observed. While the greatest increase was observed for GFAP, changes in UCH-L1 and NFL were statistically significant (Bramblett et al., 2021). Additionally, after 7 days of optic nerve compression, flash visual evoked potentials (fVEP) in the crushed eye were completely flat, while those in the uncrushed control group were normal, indicating a loss of visual function in the former. The axonal transport of cholera toxin subunit B (CT-B) had almost completely disappeared after compression, further indicating a loss of normal physiological function in the optic nerve.

The diagnosis of ONI is mostly based on history and imaging, but imaging evidence is usually not detected for several days. However, the above results suggest that levels of certain biomarkers increase immediately before extrusion and 1 h after the injury. These biomarkers may aid in the early diagnosis of ONI and prompt selection of the appropriate treatment, which may in turn lead to rapid attenuation of RGC and axon loss.

## 2. Treatment of ONI

### 2.1. Traditional treatment

For optic nerve damage due to glaucoma, the most common treatment is intraocular pressure regulation using drugs, lasers, and surgery. Intraocular pressure is currently the only controllable and measurable independent risk factor for ONI. However, lowering intraocular pressure does not completely prevent the lesion from developing (Jammal et al., 2021). Surgery and steroids can be used for TON caused by fracture or hematoma compression. However, these treatments usually only prevent the condition from progressing, while the resulting loss of RGCs and axons is irreversible (Yu-Wai-Man and Griffiths, 2011, 2013).

### 2.2. Gene therapy

Recent discoveries related to various signaling pathways, trophic factors, and inflammatory factors involved in ONI progression and protection may aid in the development of effective gene therapies that can increase the number of surviving RGCs and promote axonal regeneration.

**Figure 1:**
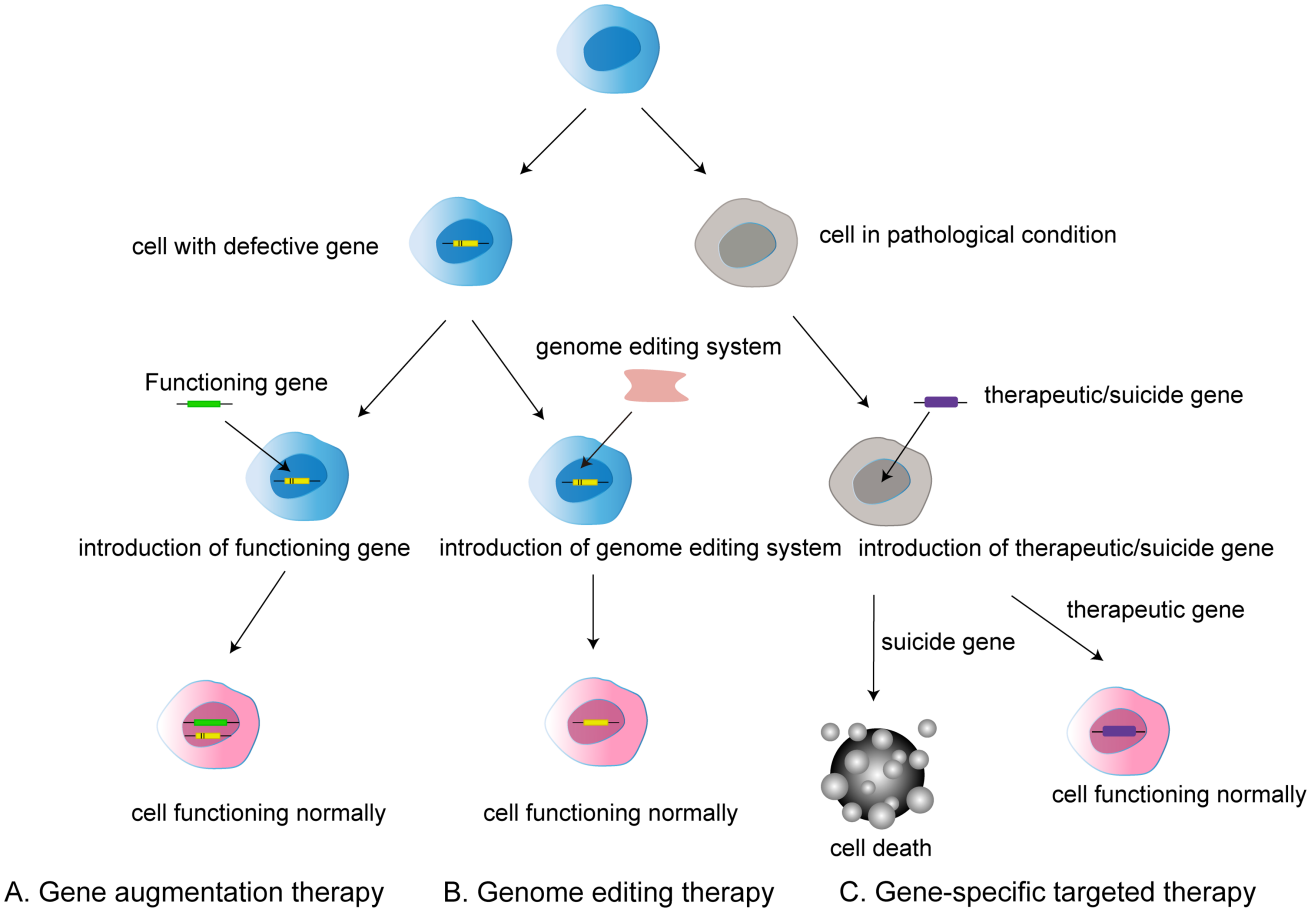
Three types of gene therapy: **(A)** Gene augmentation therapy involves introducing a functional gene into a cell that has a defective gene in order to allow the cell to function normally. **(B)** Gene editing therapy refers to the use of gene editing system to modify faulty genes into normal functioning cells. **(C)** Gene-specific targeted therapy refers to the introduction of therapeutic or suicide cells into a target cell with a disease condition to induce either death or normal function.

Gene therapy refers to the introduction of exogenous genetic material, such as DNA, RNA, siRNA, or miRNA, into cells by means of viral or non-viral vectors to regulate or replace the function of a specific gene (Campbell et al., 2016). The eye is an optimal target organ for gene therapy because of its high accessibility, relative immune privilege, and relative distance from the other organs (Boye et al., 2013). Accessibility is reflected in the fact that genetic material can be delivered to the retina during visual microsurgery. Because of the optical clarity, imaging and functional tools can easily be used to quantify the safety and efficacy of gene therapy in the eye (Zysk et al., 2007; Boon et al., 2008). Further, the blood–retina barrier prevents the transport of immune cells from the systemic circulation to the eye and suppresses inflammation, reflecting the relative immune privilege of the retina. This barrier prevents genetic material from leaking into the system circulation, allowing the expression of therapeutic genes to be localized to the eye. In addition, the target cells in the retina, such as photoreceptors and cells of the retinal pigment epithelium (RPE), do not divide, which can help to retain the long-term effects of gene therapy. Thus, gene therapy offers the possibility to target, localize, and consistently deliver therapeutic genetic material to specific intraocular sites (DiCarlo et al., 2018; Lee et al., 2019; Figure 1).

#### 2.2.1. Gene augmentation therapy

In general, gene therapy approaches can be divided into gene augmentation therapy, gene specific targeting, and genome editing. Gene augmentation therapy refers to the introduction of the functioning gene into the host genome to compensate for a faulty gene. The aim is to replace the missing and dysfunctional protein using functional gene expression. This approach offers the possibility of treatment for many previously incurable genetic diseases (Samaridou et al., 2020).

#### 2.2.2. Gene-specific targeted therapy

Gene-specific targeted therapy refers to the introduction of genetic material such as DNA or RNA into the diseased cells, in which therapeutic or suicide genes are used to specifically promote normal cell function or cell death. Such treatments are promising for non-genetic diseases and autosomal dominant genetic diseases given the ability to alter related genes or molecular pathways (Goswami et al., 2019; Lee et al., 2019).

#### 2.2.3. Genome editing therapy

Traditional genome therapy is limited in that it adds a functional gene rather than removing the faulty gene from the host genome, meaning that the expression of a faulty gene can still affect the outcome of gene therapy. However, genome editing can be used to radically modify faulty genes.

##### 2.2.3.1. Mechanics of gene editing therapy

Genome editing or correction therapy refers to the introduction of a genome editing system into target cells. It is a major means of system modification in eukaryotes. Research has indicated that DNA repair pathways can be stimulated through homology-directed repair (HDR) and non-homologous end-joining (NHEJ) using sequence specific endonucleases to generate double-strand breaks (DSBs), which immensely increase the rate of gene modification in the desired sequence (Rouet et al., 1994). HDR refers to the repair of DSBs in a custom DNA template-dependent manner that contains the desired sequence. On the contrary, NHEJ is a form of DNA repair that does not require a template that can be used to cause insertions or deletions, ultimately leading to genetic mutations.

##### 2.2.3.2. Endonucleases in genome editing

**Figure 2:**
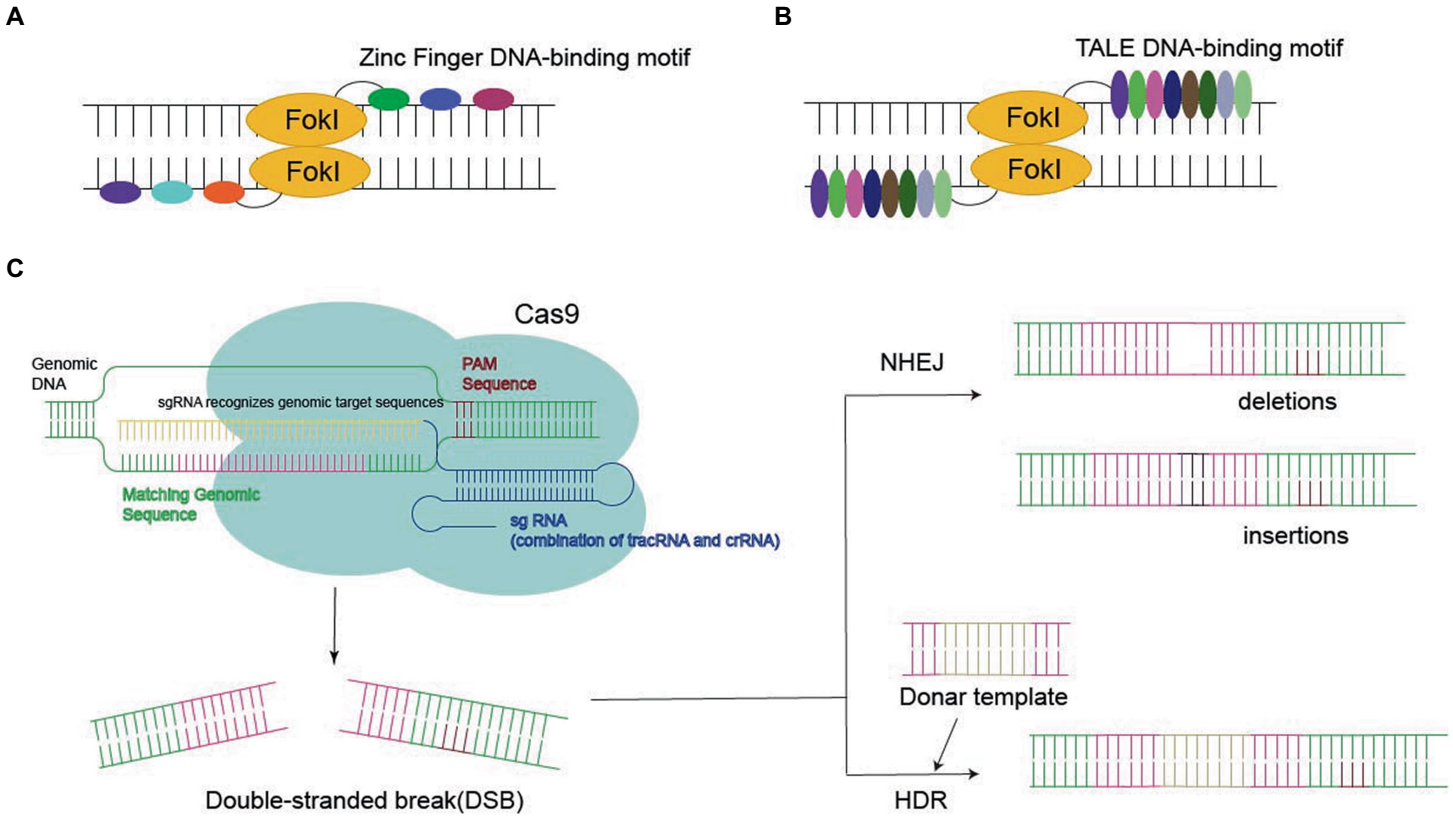
Three nucleases required for gene editing: **(A,B)** Zinc Finger DNA-binding motif or TALE DNA-binding motif bind to FokI in the target sequence, eventually leading to double-stranded break (DSB). **(C)** CRISPR-Cas9 systems generate the DSB, leading to gene deletion or insertion by homology-directed repair (HDR) or non-homologous end joining (NHEJ).

Genome editing requires various types of endonucleases, such as zinc finger nucleases (ZFNs), transcription activator-like effector nucleases (TALENs), and Clustered Regularly Interspaced Short Palindromic Repeats (CRISPR)/Cas9 (Miller et al., 1985; Smith et al., 2000; Mahfouz et al., 2011; Joung and Sander, 2013; Doudna and Charpentier, 2014; Hsu et al., 2014). These endonucleases are important for ensuring the programmability of DNA-binding domains, which are derived from zinc finger and transcriptional activator class effector proteins. As a consequence, the DNA binding specificity and affinity of zinc finger and transcription activator-like effector (TALE) proteins determine the success of the associated genetic alterations (Figure 2).

###### 2.2.3.2.1. Zinc finger nucleases

Zinc finger protein is a transcription factor with a zinc finger domain. It contains an unnatural array of more than three zinc fingers, which is the key to its application for specific DNA recognition. ZFN reflects the fusion of the ZF-binding domain to the Fokl nuclease domain, which recognizes the target site *via* sequence-specific protein–DNA interactions and cleaves adjacent sequences in DNA, finally leading to DSBs (Miller et al., 1985; Smith et al., 2000; Urnov et al., 2010; Lam et al., 2011; Carlson et al., 2012).

###### 2.2.3.2.2. Transcription activator-like effector nucleases

Transcription activator-like effectors contain DNA-binding domains consisting of a series of 33–35 amino acid repeat domains. Modular TALE repeats are linked together to recognize successive DNA sequences. TALEN reflect the fusion of the TALEs DNA-binding domain into the Fokl endonuclease fusion domain (Reyon et al., 2012). In contrast to ZFNs, each TALE repeatedly specifies one base pair, which makes it possible to select specific DNA sequences (Cermak et al., 2011; Joung and Sander, 2013; Guilinger et al., 2014).

###### 2.2.3.2.3. Clustered regularly interspaced short palindromic repeat-Cas9

Recently, CRISPR-Cas9 has become the most popular approach to site-specific genome editing (Govindan and Ramalingam, 2016). Unlike ZFNs and TALENs, CRISPR-Cas9 is a nucleotide-oriented genome editing approach that can be customized to specific DNA sequences and induce DSBs by altering single guide RNA (sgRNA; Hsu et al., 2014). SgRNA contains a 20-base pair region that binds to homologous DNA strands. Cas9 binds to DNA and produces a blunt-cut three-base set upstream of the original protospacer adjacent motif (PAM), the three-nucleotide sequence required for Cas9 binding (Jinek et al., 2012). Targeted gene insertion, mutagenesis, and gene correction can be achieved *via* HDR under the guidance of the donor DNA template to generate the desired sequence replacement at the DSB site.

The CRISPR system is an adaptive immune mechanism derived from archaea and many species of bacteria (Garneau et al., 2010). Based on the differences between their components and mechanisms, CRISPR can be divided into two systems. In class I systems, which include types I, III, and IV, RNA-guided target cleavage requires large complexes of multiple effector proteins. In class II systems, which include types II, V, and VI, only one RNA-guided endonuclease is required to mediate the cleavage of genetic material (Zetsche et al., 2015).

The CRISPR system provides a complete immune response to invading exogenous DNA in three phases. The first phase is also known as the acquisition phase. After exogenous plasmids or phages invade bacteria or archaea, the CRISPR system inside these prokaryotes incorporates exogenous DNA fragments (called proto-spacer sequences) as spacers between CRISPR RNA (crRNA) repeats into the original CRISPR motif by incorporating some titin Cas proteins.

The second phase is known as the expression phase. After the acquisition, the invader’s DNA sequence is inserted between the repetitive sequences of the CRISPR array into a new spacer sequence. This array is transcribed to obtain a complementary sequence of repetitive and spacer sequences (called pre-crRNA). At the CRISPR-Cas9 locus, there is another sequence before the Cas operon, which is transcribed separately to obtain a non-coding trans-activated RNA (tracrRNA), which hybridizes with the crRNA complex sequence and is important for crRNA processing. crRNA and tracrRNA form a complex that specifically recognizes genomic sequences, and this recognition complex can form sgRNA by fusing crRNA and tracrRNA sequences. Cas9 binding and Cas9-mediated targeting lead to cleavage. If the bacterium or archaea eventually survives, then the bacterium/archaea will translate its CRISPR array and associated proteins during the next invasion of exogenous DNA (Deltcheva et al., 2011; Hsu et al., 2014).

The third phase is known as the interference phase. At the end of the expression phase, pre-crRNAs are sheared and modified into mature crRNAs, each of which contains only a spacer sequence that wants to match the original spacer sequence of the exogenous DNA. At the time of the second invasion, the crRNA creates a match with the original spacer sequence on the exogenous DNA and activates the crRNA to bind to the Cas protein to form the RNA-Cas protein complex, which recognizes the appropriate target and shears the invading DNA, blocking the transcription of the exogenous DNA and thus protecting the host cell from infection. The presence of a sequence-specific PAM near the crRNA target site in the invasion genome and the absence of the PAM sequence at the CRISPR site in the host genome protects it from self-cleavage in the CRISPR system.

Cas9 nuclease is a naturally evolved RNA-directed nuclease derived from *Streptococcus pyogenes*. This endonuclease cleaves target DNA in class II CRISPR systems and it is the most widely used regulatory enzyme in genome editing involving Cas proteins (Gasiunas et al., 2012).

Cas9 has a typical two-lobe structure consisting of a nuclease (NUC) lobe and an alpha helix recognition (REC) lobe. The NUC lobe contains the HNH nuclease structural domain, the RuvC-like nuclease structural domain, the pam-interacting (PI) structural domain, and an evolutionarily divergent wedge structural domain (WED). The REC lobe comprises three alpha helix structural domains (Hel-I, II, III). RuvC and the HNH nuclease structural domains cleave DNA double strands, separately. The PI structural domain interacts with the PAM region of DNA through base-specific interactions and contributes to the DNA-targeting specificity of Cas9. The WED region is important for orthogonal recognition of the sgRNA scaffold and it interacts with the backbone of the PAM region (Sternberg et al., 2014; Ran et al., 2015).

Cas9-mediated genome editing is achieved through two steps: DNA cleavage followed by DNA repair. It depends mainly on two nuclease domains on the NUC lobe to function. The role of the HNH nuclease structural domain is to cut the ssDNA (target strand) paired with the gRNA, while the RuvC-like nuclease structural domain cleaves the complementary strand of the target strand.

To recognize and cleave DNA Cas9 must bind to gRNA and form a functional Cas9–RNA complex. The Cas9–sgRNA complex recognizes its DNA targets through Watson–Crick base-pairing interactions between sgRNA and target DNA and PAM interactions between Cas9 and sgRNA near the target site. Binding of the Cas9–sgRNA complex induces cleavage within the base-paired region. Thus, with only about 20 nucleotide regions in the custom sgRNA paired with the DNA sequence of interest, Cas9 can essentially be repositioned to any genomic locus containing a PAM sequence, making it an easily programmable platform for specific genomic targeting (Hsu et al., 2014).

Clustered regularly interspaced short palindromic repeat-Cas9 genome editing has already been used to correct defective genes *in vitro*. Genome editing has been shown to correct Fanconi anemia in fibroblasts from human patients (Osborn et al., 2015). Mice with a dominant mutation in the *Crygc* gene that leads to cataracts can be rescued by coinjection of Cas9 mRNA and single-channel RNA targeting the mutant allele into the zygote. CRISPR has also been used to localize the correction sequence to the endogenous *CFTR* genomic locus to accurately correct the mutation and treat pulmonary genetic diseases caused by *CFTR* mutation (Firth et al., 2015).

###### 2.2.3.2.4. Other RNA-guided endonucleases in the CRISPR system

Recently, Zetsche et al. found that Cpf1 mediated RNA-directed target gene cleavage in a class 2 V-type system. Cpf1-guided DNA cleavage is guided by crRNA only and does not require tracrRNA. In addition, Cpf1 uses a different PAM from that characterizing Cas9 and generates interleaved DSBs. Sequence analyses have revealed that Cpf1 contains only an RuvC-like structural domain and lacks the HNH nuclease structural domain found in Cas9 (Zetsche et al., 2015).

### 2.3. Vectors in gene therapy

Therapeutic genes can be transferred to target cells *via* physically or chemically mediated delivery or by using viral vectors (Trapani et al., 2014). Viral vectors are a commonly used tool in molecular biology, based on the principle that viruses have a molecular mechanism to transport their genome into other cells for infection. Due to viral diversity and the high complexity of the host organism, only a few species such as adenoviruses, AAV, lentiviruses and retroviruses can be modified as vectors for gene therapy (Bulcha et al., 2021).

Lentiviruses and AAV have a better safety profile than adenoviruses and retroviruses, and both are used in greater numbers in clinical trials. The former is the main vector for chimeric antigen receptor T-cell therapy (CAR-T therapy), while the latter is used more commonly in gene therapy (Table 1).

**Table 1:** Comparison between two common vectors used in the clinic: Lentiviruses and adeno-associated virus (AAV).

characteristic	Lentiviruses	AAV
Genome length	9 kB	4.8 kB
Pathogenicity	Yes	None
Integration into the host genome	Yes, integrated into host chromosome	None, survives as plasmid in host
Usage Scenarios	Suitable for *in vitro* applications	Suitable for *in vivo* applications
Other characteristics	Can infect non-dividing cells	Low immunogenicity and hepatotoxicity

Adeno-associated virus systems are currently the most widely used vector systems in ocular gene therapy. An AAV is a non-enveloped, single-stranded DNA virus that consists of an icosahedral protein capsid of approximately 26 mm in diameter and a single-stranded DNA genome of approximately 4.8 kb. AAV belongs to *Dependoparvovirus,* which means AAVs need a helper virus to replicate (Aponte-Ubillus et al., 2018). The helper viruses are always adenovirus and herpes simplex viruses. Their genome contains four main open reading frames (ORF). The first ORF is called the rep gene, which is responsible for encoding four proteins required for viral replication: rep78, rep68, rep52, and rep40 (Linden and Berns, 2000). The second ORF is the cap gene, which encodes the viral capsid subunits VP1, VP2, and VP3 through selective splicing and translation of different start codons. The third and fourth are the assembly-activating protein (AAP) and membrane-associated accessory protein (MAAP), which are nested sub-genomic mRNAs (Sonntag et al., 2010; Ogden et al., 2019). AAVs have two T-type inverted terminal repeat sequences (ITRs) on both sides of the genome, which mainly act as the starting point of viral replication and packaging signal, respectively (Lusby et al., 1980).

Adeno-associated viruses are non-pathogenic, non-integrating, replication-deficient, and non-immunogenic (Aponte-Ubillus et al., 2018). AAVs efficiently transduce a variety of different cell types, including all key retinal cell populations (Naso et al., 2017). Through a series of genetic modifications, the wild-type AAV can be transformed into recombinant AAV (rAAV). rAAV eliminates the rep and cap genes from wild-type AAV and retains only the ITR sequences responsible for directing the replication and packaging the viral vector genome, meaning that it integrates well into the host cell genome and reduces the risk of cellular mutations (Smith, 2008).

More than a dozen common serotypes and hundreds of virus variants of AAV have been identified, and the main differences between them are differences in the capsid protein gene. The differences in the encoded structural proteins lead to different efficiency of infection in different tissues and cells by different serotypes of AAV. AAV2 is used in research most frequently because it was the first one to be fully sequenced and characterized (Samulski et al., 1982).

In recent years, rAAV has become a major platform for the delivery of *in vivo* gene therapy. The first rAAV gene therapy product was uniQure’s alipogene tiparvovec (also named Glybera), which was approved by the European Medicines Agency in 2012 for the treatment of lipoprotein lipase deficiency.

In 2018, Luxturna was approved for marketing by the European Commission as a drug for the restoration and improvement of vision in pediatric and adult patients with loss of vision due to biallelic *RPE65* gene mutations but who retained a sufficient number of surviving retinal cells. *RPE65* mutation impairs the activity of the protein in the photopigment cycle, leading to the death of photoreceptors and thus affecting vision. Gene enhancement treatment *via* subretinal injection of an AAV vector with the addition of a normal copy of the *RPE65* gene has been shown to achieve improvements in visual acuity (Redmond et al., 2005; Smalley, 2017).

Although viral vectors such as AAV have made initial progress in gene therapy, there are still some limitations that need to be resolved. Among them, vector immunogenicity is the biggest challenge of virus-based gene therapy (Shirley et al., 2020; Bulcha et al., 2021). By understanding these immune responses, vector design will be improved in the future to enable better targeted immunomodulation. Viral vectors are recombinant molecules composed of eukaryotic transgenes and viral capsids, which are not viruses and cannot direct the synthesis of viral proteins. However, the human immune system does not recognize the difference between the recombinant capsid and real viral capsid, and the immune response to the vector is influenced by the previous exposure to the wild-type virus. In the case of AAV, strategies for overcoming AAV humoral immunity are being investigated. The current approach to neutralizing antibody (NAB) to AAV is used to exclude patients with AAV antibodies. However, this approach is not feasible because it involves a broad population. The methods that may be currently available are selection of subjects with low to undetectable anti-AAV NAB, use of high dose vectors, use of empty shell adsorbed anti-AAV antibodies, etc. These methods can be used either individually or in combination (Mingozzi and High, 2011). In addition to immunogenicity limitations, the performance of AAV vectors evaluated *in vitro* is unsatisfactory, and *in vivo* AAV transduction does not necessarily reflect the same as observed *in vitro*. Furthermore, it is difficult to obtain the rAAV carrier without impurities, and finding new strategies to increase its production remains an open question. Lentiviral vectors have become vectors of choice for transgene delivery due to their low genotoxicity, but these vectors have been shown to cause abnormal splicing of cellular transcripts, which remains a challenge to be addressed in the future (Bulcha et al., 2021).

### 2.4. Cell therapy

As described later, the use of gene therapy to induce RGC’s survival and regeneration has improved, but recent studies have shown that the axons cannot be regenerated over long distance and cannot be re-established in the visual pathway (Li et al., 2017a). Gene therapy has provided strong support for solving this problem, such as activating mammalian target of rapamycin channels and knocking out PTEN (Li et al., 2015; Lim et al., 2016). In addition to gene therapy, cell therapy has also contributed to long-distance regeneration of axons. Cell therapy is used to replace the lost cells with cells that have a multiple differentiation potential. Retinal progenitor stem cells (RPCs), human pluripotent stem cells (PSCs), and mesenchymal stem cells (MSCs) have been confirmed to differentiate into neurons in clinical trials (Wang et al., 2007). HPMSC under the tenon transplant has a protective effect on the RGCs through clinical trials (Sung et al., 2020b). Two characteristics of hPMSC: suitable to acquire *in vitro* and immune tolerance, showing strong clinical potential (Li et al., 2017b). Studies have found that the treatment of MSCs from human Wharton’s jelly (HWJ-MSCS) can cause sustained survival of RGCs, significant long-distance axon regeneration, and partial recovery of synaptic function (da Silva-Junior et al., 2021). Some scholars have studied the synergy between gene therapy and cell therapy. The overexpression of pigment epithelium-derived factor (PEDF) has a neuroprotective effect on RGC after optic nerve compression, but cannot promote axon growth. Co-treatment with PEDF and hMSC shows significant axon growth (Nascimento-Dos-Santos et al., 2020). The combination of the two may offer new hope for re-establishing the connection between the eye and the brain. The site of action of cell therapy is also a concern in clinical treatment. The intravitreal injection is currently common, but it may be that the retinal barrier prevents cells from migrating to the correct location, and while subretinal transplantation can solve this problem, it requires excellent surgical skills (Coco-Martin et al., 2021). Both have been reported to have adverse effects such as proliferative vitreoretinal bands (Bai et al., 2017; Özmert and Arslan, 2020). Gene therapy and cell therapy are both potential treatments, and genetically modified stem cells may be the focus of future research.

## 3. Gene therapy for ONI

### 3.1. Brain-derived neurotrophic factor

In all mammals, brain-derived neurotrophic factor (BDNF) is the most widely distributed neurotrophic factor and plays an important role in the normal growth, development, and plasticity of nerves as well as in neuroprotection (Kowiański et al., 2018). BDNF can be produced by neurons such as RGCs, amacrine cells, retinal glial cells, and photoreceptors (Telegina et al., 2019). Through the optic nerve, BDNF circulates in the retina and brain (Feng et al., 2016). BDNF can regulate laminar refinement in the dendrites of RGCs during visual development, contributing to the proper formation of the retinal structure (Liu et al., 2007). BDNF is able to exert its functions by binding to its receptor, tropomyosin receptor kinase B (TrkB; Mysona et al., 2017). Animal models of ONI are generated by inducing elevated intraocular pressure (IOP) using microbead injections. Studies have reported significant attenuation of RGC loss in injured rats treated with AAV2-BDNF compared with injured untreated rats (Wójcik-Gryciuk et al., 2020). In mice subjected to optic nerve crushing, the same AAV2 TrkB-2A-mBDNF vector resulted in significantly greater attenuation of RGC loss than BDNF treatment alone, resolving the downregulation of *TrkB* caused by the long-term injection of *BDNF* (Frank et al., 1996; Osborne et al., 2018). Another study concluded that high glucose level is toxic to RGCs (Oshitari et al., 2010). The decrease in the number of apoptotic cells in the HG medium containing BDNF is correlated with the suppression of caspase-9 and caspase-3 activities. The active form of caspase-3 is the executioner caspase that activates apoptosis in retinal cells. However, cells incubated with neurotrophic factors (e.g., BDNF) exhibit significantly lower levels of apoptosis than those in the control group.

### 3.2. Histone acetyltransferases (HATs) and histone deacetylases (HDACs)

DNA methylation and histone post-translational modifications (PTMs) are two major mechanisms of epigenetic regulation of gene expression. Histone acetylation is a major modification affecting gene transcription and is dynamically regulated by both HATs and HDACs. HATs acetylate lysines in histones, leading to the loosening of chromatin structure and promoting gene expression. In contrast, HDACs compact chromatin and inhibit general gene transcription (Shen et al., 2015). Evidence indicates that neurodegenerative states are associated with disturbances in histone acetylation. HATs are often degraded under pathological conditions such as intraocular hypertension, optic nerve damage, ischemia, and hypoxia. However, the activity of HDACs is significantly enhanced, which leads to a relative excess of deacetylation activity. This in turn promotes the production of heterochromatin and silencing of specific genes, which leads to the physiological dysfunction of nerve cells (Saha and Pahan, 2006).

Schmitt et al. (2014) achieved specific knockout of *HDAC3 via* transfection of AAV-2, following which they established an optic nerve crush model. Their experiments demonstrated that the knockdown of *HDAC3* significantly improved the characteristics of ONC-induced nuclear atrophy and reduced the death of RGCs. Zhang et al. subcutaneously injected valproic acid (VPA) and sodium butyrate (SB), a histone deacetylase inhibitor, 14 days before ONC (Zhang et al., 2012). Results from the sacrificed mice indicated that VPA and SB ameliorated the decreased levels of ERG a-wave and b-wave amplitudes and attenuated caspase-3 activation in RGCs, which was accompanied by phosphorylation of threonine kinase (AKT) and extracellular signal-regulated kinase (ERK). It is believed that VPA may hinder the apoptosis in RGCs by activating the BDNF–TrkB signaling pathway and inhibiting HDACs. Sun Songmei et al. evaluated the neuroprotective effect of intravitreal injection of liposomes loaded with the HDAC inhibitor TSA in a mouse model of ONC. The authors reported that the liposomes reached the medial retina after injection, significantly reducing the expression of reactive glia, hyperplasia, and apoptosis of RGCs (Sung et al., 2020a). In summary, HDACs are already therapeutic targets for neurodegenerative diseases. Their considerable neuroprotective effects in models of ONI lay the foundation for future inhibition of HDACs to regulate the survival of RGCs.

### 3.3. Phosphatase-tensin homolog (PTEN) and suppressor of cytokine signal transduction 3 (SOCS3)

Phosphatase-tensin homolog (PTEN) and SOCS3 are regulators of signal transduction pathways that control cell proliferation and survival, cell migration, and genome stability. Studies have shown that RGC survival is higher in mice with *PTEN* or *PTEN* and *SOCS3* deletion than in those with simple optic nerve injury or simple knockout of *SOCS3*. In these two groups, retention and regeneration of dendrites and axons could be observed after optic nerve compression (Mak et al., 2020). Phosphatase-tensin homolog (PTEN) may play a greater role in promoting optic nerve regeneration. Studies have also reported a high degree of axonal regeneration in both the corticospinal tract injury model and the adult mouse ONI model, which may be related to the specific knockdown of *PTEN* in mice, since this increases the activity of the mTOR pathway (Huang et al., 2018). Another study demonstrated that, 4 weeks after ONI, simultaneous knockdown of *PTEN* and *SOCS3* resulted in stronger axonal regeneration than single knockdown of either *PTEN* or *SOCS3* alone (Li et al., 2015). These results suggest that deletion of *PTEN* or *SOCS3* can protect the optic nerve, promoting the proliferation of RGCs and the regeneration of optic axons and that the effects are more significant when both genes are knocked out simultaneously.

### 3.4. RNA

Excessive astrocyte activation and glial scar formation are detrimental to axon regeneration after ONI. MicroRNA-21 (miR-21) is a negative regulator of gene expression. One group has reported that inhibition of miR-21 can decrease protein levels of EGFR/PI3K/AKT/mTOR, attenuating excessive astrocyte activation and glial scar formation after optic nerve crush. Intravitreal injections of an miR-21 mimic (agomir) and an miR-21 inhibitor (antagomir) were given immediately after optic nerve crush (Li et al., 2018). Injured rats treated with antagomir had significantly more axons than those in the negative control group. Further, fVEP amplitude was significantly higher in injured rats treated with antagomir than in those treated with agomir. These results suggest that inhibition of miR-21 can induce an environment more conducive to axonal regeneration and functional recovery following ONI.

One study found that RGCs die by apoptosis through cleavage of caspase-3, -8, and -9 in a preclinical glaucoma model of optic nerve crush in adult rats (Tawfik et al., 2021). The greatest increase was observed for caspase-3, with the highest levels observed during the primary wave of RGC loss, although they remained high during the RGC degeneration phase (Almasieh et al., 2012; Sánchez-Migallón et al., 2016). Gene therapy can block caspase-3 gene expression and the loss of RGCs *via* the use of non-viral siRNA-nanoparticles. The use polybutylcyanoacrylate nanoparticles (PBCA-NPs) has a significant advantage: crossing barriers. Indeed, PBCA-NPs can transfer siRNA across the blood-retinal barrier, which is not possible when using nanoparticles made of other materials (Tawfik et al., 2021). At the same time, scholars have also found that siRNA wrapped in PBCA-NPs can inhibit caspase-3 expression through the inner boundary membrane by vitreous injection, which cannot be achieved by direct vitreous injection of siRNA (Tawfik et al., 2021). Direct use of siRNA has immune recognition, easy degradation, and other shortcomings (Hayreh, 2020). AAV is still the most commonly used tool in gene editing, but the adverse reactions are still prominent such as capsid immunogenicity, antibodies against viral capsid even if the concentration is very low, neutralizing AAV carrying the target gene, greatly limiting the clinical use of the same serotype viral vector (Taha et al., 2022). The recent popular CRISPR-Cas9 also has the problem of immunogenicity, and Cas9 can induce cellular immune responses in mice. Empty AAV9 without Cas9 did not cause any significant cell infiltration (Chew et al., 2016). Delivering siRNA by PBCA-NPs reduces degradation and immune recognition, providing more stable gene expression (Tawfik et al., 2021; Taha et al., 2022). As such, gene therapy with siRNA-nanoparticles represents a promising research avenue for patients with central visual system damage and other neurological disorders.

### 3.5. Ciliary neurotrophic factor and CCL5

Recent studies have found that Ciliary neurotrophic factor (CNTF) gene therapy can induce optic nerve regeneration in animal models, making it the drug of choice for treatment of a variety of eye diseases, while C–C motif chemokine ligand 5 (CCL5) can induce extensive axon regeneration and mediate the effect of CNTF gene therapy (Xie et al., 2021). CNTF is therefore one of the most important neurotrophic factors in optic retinal degenerative diseases. It has been found that CNTF has neurotrophic function in RGCs (Fischer and Leibinger, 2012; Wen et al., 2012). In the rat model of optic nerve axotomy, a single injection of CNTF protein into the vitreous can significantly protect RGCs, while BDNF has no protective effect (Mey and Thanos, 1993). Moreover, in the ONC model of CNTF gene therapy mediated by AAV vector, the RGC survival rate was greatly improved compared with the control group (Leaver et al., 2006). However, although CNTF gene therapy promoted regeneration, recombinant CNTF (rCNTF) was less effective, possibly because rCNTF significantly increased SOCS1 and SOCS3 mRNA and protein levels in RGCs (Smith et al., 2009). It was found that CNTF gene therapy exacerbated the inflammatory response to viral-mediated gene therapy, significantly increasing CCL5 expression in immune cells and retinal Müller cells. Neutrophil depletion, overall knockout (KO) or RGC-selective deletion of the CCL5 receptor CCR5 or CCR5 antagonists inhibited the effects of CNTF gene therapy, whereas recombinant CCL5 (rCCL5) promoted axonal regeneration and increased RGC survival. CCL5 is a chemokine that promotes recruitment of immune cells such as T cells, monocytes/macrophages, and eosinophils by binding to one or more G-protein-coupled receptors. The absence of CCL5 leads to the disorganized morphology of RGC dendrites and non-secretory cells. Therefore, CCL5 is an effective agent for optic nerve regeneration and RGC survival. These findings provide insights for understanding the mechanism of CNTF gene therapy and guiding clinical trials (Xie et al., 2021).

## 4. Deficiencies of gene therapy

As mentioned above, gene therapy involves the introduction of exogenous normal genes into target cells through technical means to correct or compensate for the diseases caused by defective or abnormal genes, which aid in the treatment of previously incurable diseases such as cancer and immunodeficiencies. Over the past decades, substantial progress has been made in the field of ophthalmology. However, gene therapy is still an imperfect treatment method, and there are many problems that must be addressed before its clinical application.

### 4.1. Defects in gene editing techniques

Use of the CRISPR system for gene editing has many limitations. First, the CRISPR system is not completely accurate. Given that there are 3 billion base pairs in addition to the target gene, the potential for misalignment is huge. When modifying the human genome, we cannot afford to make any mistakes that could lead to cancer, apoptosis, or other unexpected negative effects. CRISPR researchers are working on effective ways to solve this problem, such as modifying RNA to improve the accuracy and building artificial Cas proteins. However, clinical applications of CRISPR are still a long way off.

Insertional mutagenesis is one of the major safety concerns of CRISPR. Insertional mutagenesis refers to the translocation of gene material, which disrupts chromatin or gene structure, leading to changes in the transcription, regulation, or coding sequence of the gene. In particular, when integrated vectors are used, they integrate into active regulatory or transcriptional regions of genes. If such insertion upregulates the expression of endogenous proto-oncogenes, it may lead to tumorigenesis (Cavazza et al., 2013).

### 4.2. Host immune response

Studies have reported that humans may exhibit an immune response to the AAV coat, which is mediated by AAV capsid-specific CD8 + T cells to transduce the immune rejection of hepatocytes (Mingozzi and High, 2011). In humans, AAVs trigger a specific type of immunity. These factors act as part of the innate immune response, resulting in either an immediate response to proteins encoded by the viral capsid or vector, or in a more specific adaptive response that generates immune memory. As a result of this immune activation, the transduced vector cells are rapidly destroyed. In addition, humans are the natural hosts of AAV, and serum tests are highly positive for AAV vectors. This limits the broad use of AAV vectors in patients with preexisting neutralizing antibodies or memory T cells (Hareendran et al., 2013).

Many strategies have been developed to solve this problem. An anti-AAV immune response can be avoided by local delivery of AAV to immunologically privileged organs such as the eyes. If systemic delivery is necessary, transient immunosuppression can be used to create a window for AAV vector delivery, which can mitigate the immune response. Recombinant AAV vectors can also be modified to reduce cellular and humoral immunity.

In addition, some studies applying AAV-mediated gene therapy have reported that AAVs can induce local and systemic immune responses, intraocular inflammation, and decrease in initial functional improvements even when systemic anti-inflammatory treatment is performed with corticosteroids (Bainbridge et al., 2015). This is referred to as gene therapy-associated uveitis (GTAU), which can lead to the deterioration of visual acuity. AAV vector-induced retinal toxicity can also decrease ERG amplitudes and lead to retinal thinning following damage to photoreceptors and the RPE in preclinical models (Khabou et al., 2018). In addition, GTAU can be accompanied by anterior chamber inflammation, vitritis, vasculitis, mononuclear retinal, and choroidal inflammation/endophthalmitis (Ye et al., 2016; Cukras et al., 2018; MacLachlan et al., 2018; Tobias et al., 2019). As such, a significant number of patients may reject AAV gene therapy given these adverse reactions. Treatment strategies to alleviate or restrain the immune responses against AAV comprise vector-oriented and immune response-oriented approaches. Generating neutralizing antibody-resistant AAVs with empty capsids and changing antigenic epitopes are the next key directions in ONI research. In clinical trials, corticosteroids have been widely adopted for transient immunosuppression.

### 4.3. Potential effectiveness of gene therapy

The ability of gene therapy vectors to effectively transduce various cell types, such as precursor photoreceptor cells, at different stages of eye development must be considered when attempting to translate gene therapy into clinical application, as this is necessary for ensuring that the approach is useful for the desired disease-affected population. Gene therapy does not benefit a wide range of patients at present. Indeed, it can only benefit certain groups of patients, especially those with only minor pathological changes or those in the early course of the disease. Leber congenital amaurosis (LCA), or early-onset retinal dystrophy, is caused by a mutation in the *RPE65* gene that disrupts the retinoid cycle and ultimately leads to severe visual impairment. It is characterized by poor vision, loss of ERG responses, nystagmus, and abnormal pupillary light reflexes. Retinal anatomy also degrades, but not completely. In one study, three young adults with RPE65-LCA underwent *RPE65* gene replacement therapy using AAV2, and visual acuity was measured before and 90 days after the intervention. In this study, all patients exhibited a significant increase in visual acuity 30 days after treatment, which was restricted to the retinal region receiving the vector, although there was no change between 30 and 90 days (Miraldi Utz et al., 2018). The reason for the failure to achieve consistent improvements in visual acuity remains unclear, although it may have been related to insufficient expression of RPE65 to effectively maintain the visual cycle and the degeneration of the retina resulting in insufficient surviving photoreceptors to meet visual demands.

### 4.4. Cost effectiveness of gene therapy

Gene therapy research and its application require substantial time and resources, representing an important barrier in clinical translation. The estimated cost of drug development for gene therapy ranges from $1.3 to $1.7 billion (Vandenberghe, 2015). This cost is likely to increase along with the development of the global economy. The cost of clinical trials is one of many important factors driving up overall costs. Martin et al. reported that the median cost of conducting a clinical trial from protocol approval to final reporting of the clinical trial was $3.4 million for a phase I trial, $8.6 million for a phase II trial, and $21.4 million for a phase III trial (Martin et al., 2017). The high cost of gene therapy will eventually raise a social ethical question, namely whether the introduction of such expensive gene therapy requires the expansion of medical insurance coverage, and whether it is beneficial or unfavorable to the development of the economy. Promoting the advantages of gene therapy, changing the traditional funding mechanism, developing a one-off sustainable treatment modality, and actively developing genome editing technology as the biotechnology industry expands may help to solve the funding problem (Hall and Carlson, 2014).

## 5. Conclusion

Damage to the optic nerve is generally considered irreversible. However, some patients exhibit different degrees of visual recovery after treatment, which indicates that the optic nerve can regenerate under certain circumstances. Despite great advancements in gene therapy for optic nerve protection, it is still very difficult to reproduce the results observed in animal models in clinical trials, and most injury models in animals are acute, in contrast to the chronic form of ONI commonly observed in clinical settings. While promising, several problems must be addressed before gene therapy can be applied to ONI in human patients. Methods for controlling the amount of AAV transferred must be developed. Moreover, miRNA in mice can lead to organ failure, and the risk may be much greater in human application. Exploring the mechanisms underlying ONI, developing more precise gene therapies, accelerating the safe transition between animal models and human experiments, and finding the balance between cost and output are key directions for the future of ONI research.

## Author contributions

KX and LY: conceptualization. KX, LY, and ZW: validation. YX: investigation. PL: resources. NZ: data curation. KX: writing—original draft preparation. NY: writing—review and editing. NY and YX: supervision. All authors have read and agreed to the published version of the manuscript.

## Funding

This research was funded by Hubei Key Laboratories Opening Project, grant number 2021KFY055 and Natural Science Foundation of Hubei Province, grant number 2020CFB240, and Fundamental Research Funds for the Central Universities, grant number 2042020kf0065.

## Conflict of interest

The authors declare that the research was conducted in the absence of any commercial or financial relationships that could be construed as a potential conflict of interest.

## Publisher’s note

All claims expressed in this article are solely those of the authors and do not necessarily represent those of their affiliated organizations, or those of the publisher, the editors and the reviewers. Any product that may be evaluated in this article, or claim that may be made by its manufacturer, is not guaranteed or endorsed by the publisher.

## Abbreviations

ONI: optic nerve injury
RGCs: retinal ganglion cells
TLR2: Toll-like receptor 2
Ocm: oncomodulin
SDF1: stromal cell-derived factor 1
RNFL: retinal nerve fiber layer
IOP: intraocular pressure
TBI: traumatic brain injury
UCH-L1: ubiquitin carboxy-terminal hydrolase L1
NFL: neurofilament light chain protein
fVEP: flash visual evoked potentials
CT-B: cholera toxin subunit B
RPE: retinal pigment epithelium
HDR: homology-directed repair
NHEJ: non-homologous end-joining
DSBs: double strand breaks
ZFNs: zinc finger nucleases
TALENs: transcription activator-like effector nucleases
CRISPR: clustered regularly interspaced short palindromic repeats
BDNF: brain derived neurotrophic factor
HATs: histone acetyltransferases
HDACs: histone deacetylases
PTMs: post-translational modifications
GTAU: gene therapy-associated uveitis
